# Comparison of International Normalized Ratio Measurement between CoaguChek XS Plus and STA-R Coagulation Analyzers

**DOI:** 10.1155/2013/213109

**Published:** 2012-12-24

**Authors:** Mina Hur, Hanah Kim, Chul Min Park, Antonio La Gioia, Sang-Gyu Choi, Ju-Hee Choi, Hee-Won Moon, Yeo-Min Yun

**Affiliations:** ^1^Department of Laboratory Medicine, School of Medicine and Konkuk University Hospital, Konkuk University, 120-1 Neungdong-ro, Hwayang-dong, Gwangjin-gu, Seoul 143-729, Republic of Korea; ^2^Italian Society of Clinical Biochemistry and Molecular Biology, Rome, Italy

## Abstract

*Background*. Point-of-care testing (POCT) coagulometers are increasingly being used in the hospital setting. We investigated whether the prothrombin time international normalized ratio (INR) results by CoaguChek XS Plus (Roche Diagnostics GmbH, Mannheim, Germany) can be used reliably without being confirmed with the INR results by STA-R system (Diagnostica Stago S.A.S, Asnières sur Seine, France). *Methods*. A total of 118 INR measurements by CoaguChek XS Plus and STA-R were compared using Passing/Bablok regression analysis and Bland-Altman plot. Agreement of the INR measurements was further assessed in relation to dosing decision. *Results*. The correlation of INR measurements between CoaguChek XS Plus and STA-R was excellent (correlation coefficient = 0.964). The mean difference tended to increase as INR results increased and was 0.25 INR in the therapeutic range (2.0-3.0 INR). The overall agreement was fair to good (kappa = 0.679), and 21/118 (17.8%) INR measurements showed a difference in dosing decision. *Conclusion*. The positive bias of CoaguChek XS Plus may be obvious even in the therapeutic INR range, and dosing decision based on the CoaguChek XS Plus INR results would be different from that based on the STA-R results. The INR measurements by POCT coagulometers still need to be confirmed with the laboratory INR measurements.

## 1. Introduction

High-quality anticoagulation management is necessary to keep the narrow therapeutic index medications as effective and safe as possible. Oral anticoagulation therapy should be managed in a systematic and coordinated fashion, incorporating patient education, systematic international normalized ratio (INR) testing, tracking, followup, and good patient communication of results and dosing decisions. Prothrombin time (PT) INR is fundamental to prevent bleeding complications or thrombotic events during oral anticoagulation therapy [1, 2]. The target range for INR is dependent on the clinical condition being monitored. For example, targeting an INR of 2.0 to 3.0 for patients with atrial fibrillation, deep vein thrombosis, pulmonary embolism, and heart valves on vitamin K antagonist therapy is one of the strong recommendations of the American College of Chest Physicians [3].

Point-of-care testing (POCT) coagulometers are increasingly being used in the general practice setting by primary healthcare providers and by patients and have the potential to improve management of anticoagulation therapy. However, there have been several documented limitations regarding the accuracy and precision of these devices, including greater differences compared with a standard plasma-based methodology as INRs increase above the therapeutic range [4–7]. Given that INR methods are not harmonized, when monitoring patients on warfarin it is best to keep to one method, and swapping between different laboratory methods or going from laboratory methods to POCT should be discouraged. Nevertheless, using POCT coagulometers is beneficial in that INR results are readily available using capillary blood from a fingertip or untreated venous whole blood instead of citrated venous blood for standard laboratory analyzers [8, 9]. Accordingly, the need for implementing these POCT coagulometers has increased even in the tertiary care hospitals by the clinicians as well as by the patients.

There have been limited comparisons between CoaguChek XS Plus (Roche Diagnostics GmbH, Mannheim, Germany) and STA-R automated coagulation system (Diagnostica Stago S.A.S, Asnières sur Seine, France) [10]. In this study, we compared the INR results between CoaguChek XS Plus and STA-R to know how interchangeable both INR results are and whether the CoaguChek XS Plus INR results can be used reliably for following up the patients without being confirmed or validated with the results of standard laboratory analyzer.

## 2. Materials and Methods

### 2.1. Study Population and INR Measurements

A total of 118 patients were enrolled in this study. They were 70 males and 48 females, and their median age was 68 years (range, 5–87 years). During the period between May and July in 2011, they presented to the outpatient clinic of Konkuk University Medical Center, Seoul, Korea, for the baseline screening of their coagulation system or for the routine monitoring of oral anticoagulation therapy. They were recruited from the departments of cardiovascular surgery (*n* = 50), cardiology (*n* = 42), neurology (*n* = 19), and others (*n* = 7). All blood samples were obtained in the blood collection room for outpatients by one certified phlebotomist, who had about 15-year experience for the blood collection and clinical laboratory tests. Each patient was scheduled to draw the venous blood and gave informed consent to participate in this study. Because either capillary blood or venous blood can be used for the analysis in the CoaguChek XS Plus, to avoid dual sampling, venous blood was used for the comparison. This study was approved by the institutional review board.

From a venipuncture approximately 5 mL of blood was drawn into a syringe. The 2.7 mL venous blood was put into a tube containing 3.2% buffered sodium citrate and was sent to the laboratory for the INR measurement using STA-R system. The remaining blood in the syringe was used for the INR measurement by CoaguChek XS Plus without delay. CoaguChek XS Plus was operated by the same phlebotomist. The preanalytical conditions (differences) were thought to be not influential. The CoaguChek XS Plus uses a human recombinant thromboplastin (ISI = 1.01) and employs electrochemical current detection to measure clot formation. In whole blood testing the mean coefficient of variation of the CoaguChek XS Plus PT determination was claimed to be in the range of 1.3% to 1.6% by the manufacturer. The citrated venous blood samples for STA-R were processed and analyzed immediately after collection according to the routine procedures of the laboratory. The laboratory measurements using STA-Neoplastine CI Plus kit (Diagnostica Stago S.A.S) were considered the reference standard method. 

### 2.2. Statistical Analysis

The INR measurements were analyzed using Pearson's correlation coefficient, Passing/Bablok regression analysis, and Bland-Altman plot. Bland-Altman plot was used to identify mean difference and 95% limits of agreement of the INR results between CoaguChek XS Plus and STA-R. The overall correlation and difference were compared in a total of 118 measurements and were further compared in two INR ranges (1.0-2.0 INR and 2.0-3.0 INR). Agreement of INR measurements was also assessed according to the three ranges of dosing decision (subtherapeutic, therapeutic, and supratherapeutic ranges) with cut-off values of 2.0 INR and 3.0 INR, respectively. Cohen's Kappa value was used for assessing agreement (<0.4, poor; 0.4–0.75, fair to good; >0.75, excellent). Statistical analysis was performed using MedCalc Statistical Software (version 12.3.0, MedCalc Software, Mariakerke, Belgium), and *P* values less than 0.05 were considered statistically significant.

## 3. Results

**Figure 1:**
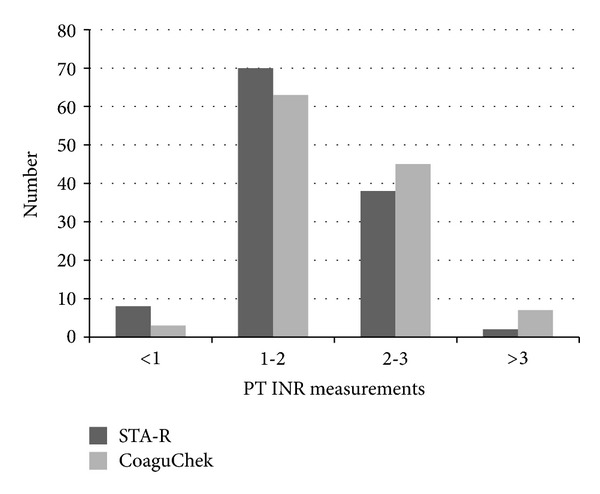
The distribution of prothrombin time (PT) international normalized ratio (INR) measurements by CoaguChek XS Plus and STA-R.

**Figure 2:**
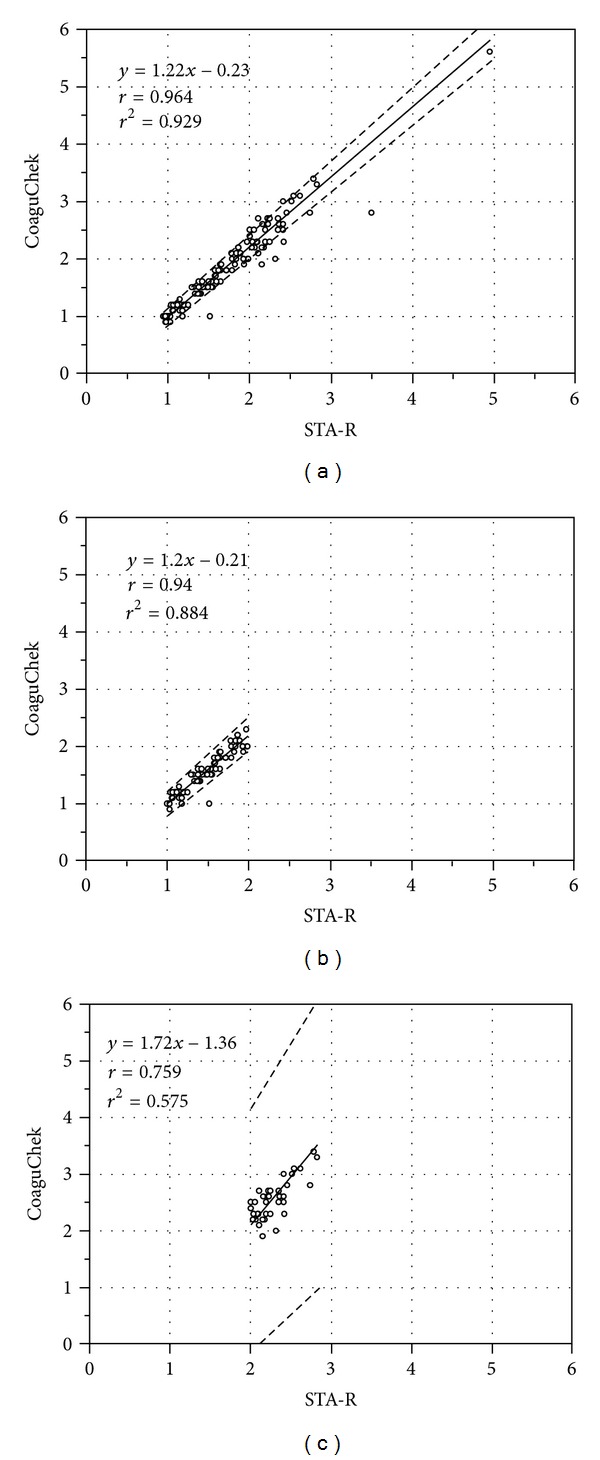
Comparison of INR measurements between the CoaguChek XS Plus and the STA-R using Passing/Bablok regression analysis. The solid lines indicate the regression lines, and the dashed lines indicate the 95% confidence interval (CI). (a) In a total of 118 measurements, Passing-Bablok regression analysis gave a slope of 1.22 (95% CI, 1.16–1.28) and an intercept of −0.23 (95% CI = −0.32–0.14). (b) In the range of INR 1.0-2.0 (*n* = 70), it gave a slope of 1.20 (95% CI, 1.11 to 1.30) and an intercept of −0.21 (95% CI, −0.35 to −0.9). (c) In the range of INR 2.0-3.0 (*n* = 38), it gave a slope of 1.72 (95% CI, 1.33 to 2.31) and an intercept of −1.36 (95% CI, −2.36 to −0.48).

Based on the STA-R system, the INR measurements ranged from 0.95 INR to 4.95 INR. The distribution of INR measurements by CoaguChek XS Plus and STA-R is presented in Figure 1. The overall correlation of the INR measurements between CoaguChek XS Plus and STA-R was excellent without significant deviation from linearity. The Pearson's correlation coefficient in all 118 measurements was 0.964 (95% confidence interval [CI], 0.948–0.975; *P* < 0.0001). When the correlation was further assessed in the ranges of 1.0-2.0 INR (*n* = 70) and 2.0-3.0 INR (*n* = 38), the Pearson's correlation coefficient was 0.940 (95% CI, 0.906–0.963; *P* < 0.0001) and 0.759 (95% CI, 0.580–0.868; *P* < 0.0001), respectively (Figure 2).

**Figure 3:**
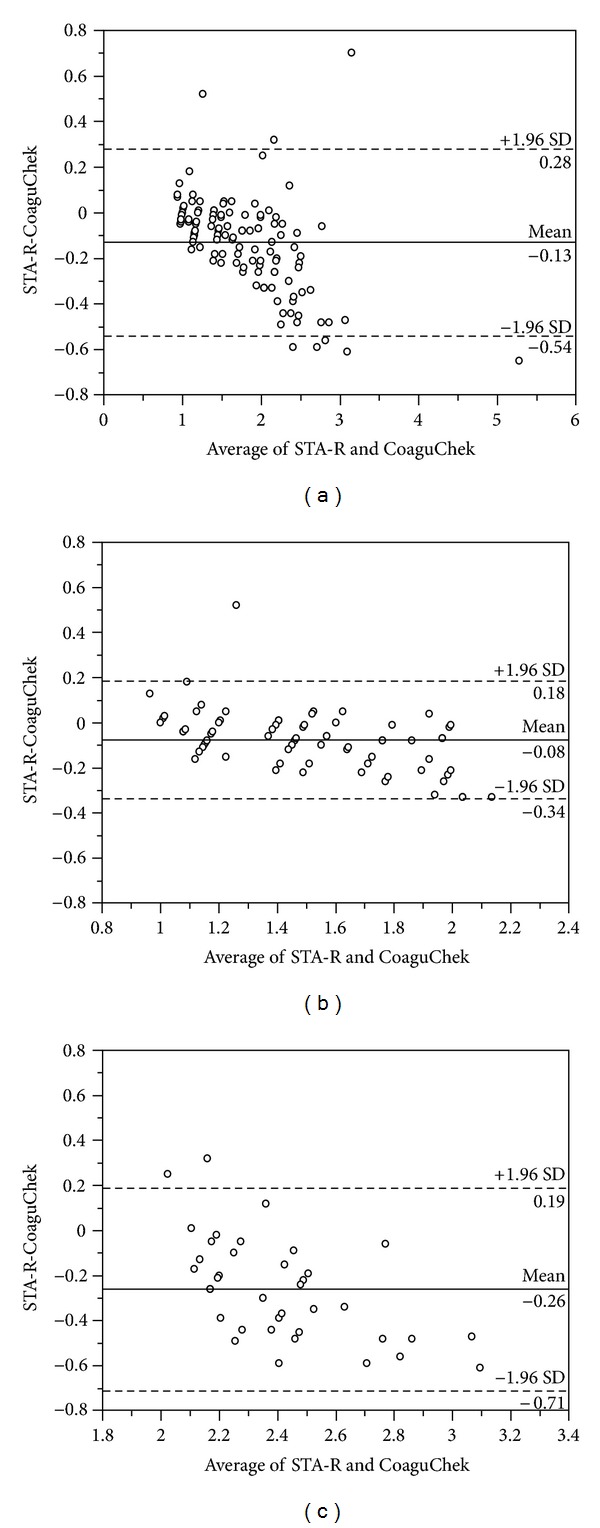
Comparison of INR measurements between the CoaguChek XS Plus and STA-R using Bland-Altman plots in a total of 118 measurements (a), in the range of INR 1.0-2.0 (b), and in the range of INR 2.0-3.0 (c). The difference between two values in the *y*-axis is plotted against the average of STA-R and CoaguChek XS Plus results in the *x*-axis. The solid lines represent the mean differences in INR measurements between the two methods, and the dashed lines represent mean difference ±1.96 standard deviation (SD).

The mean difference between the INR measurements by STA-R and CoaguChek XS Plus was −0.13 INR. For differences with 95% limits of agreement (1.96 standard deviations [SD] of the mean difference), the STA-R INR measurements differed from the CoaguChek XS Plus INR measurements by −0.54 INR to 0.28 INR. The mean difference of INR measurements tended to increase as INR values increased, and CoaguChek XS Plus exhibited increasing positive bias compared with STA-R at higher INR measurements. The mean difference of the INR measurements was −0.08 (±1.96 SD, −0.34–0.18) in the lower range (1.0-2.0 INR) and −0.26 (±1.96 SD, −0.71–0.19) in the higher range (2.0-3.0 INR), respectively (Figure 3).

The agreement of INR measurements between CoaguChek XS Plus and STA-R was further assessed according to the three INR ranges (subtherapeutic, therapeutic, and supratherapeutic ranges) related to dosing decision. The overall agreement was fair to good (kappa = 0.679; 95% CI, 0.569–0.790), and 21/118 (17.8%) INR measurements showed a difference in dosing decision between the two instruments (Table 1).

## 4. Discussion

Although there have been numerous studies on POCT coagulometers, they were all different in the study designs and statistical analyses, leading to diverse conclusions regarding the precision and accuracy of POCT coagulometers [4, 6, 11, 12]. In a recent review, the precision and accuracy of POCT coagulometers were regarded as generally acceptable for clinical use [11]. On the contrary, another systematic review did not provide robust evidence that POCT in general practice improves patient health outcomes and that it has comparable analytical quality to pathology laboratory testing. That review also stated that drawing firm conclusions are also difficult because of the different measurement technologies used for both POCT and in the laboratory [7].

The CoaguChek XS Plus system was designed for use in the professional setting, differently from the CoaguChek XS system designed for use in patient-self testing [13]. Several studies have evaluated the clinical use of the CoaguChek XS Plus system [10, 13–17]. Those studies were performed in different clinical settings using different laboratory-based tests, and only one of them compared the CoaguChek XS Plus system with the Stago coagulation system (Table 2).

Given these various and questionable conclusions in relatively limited literature on the CoaguChek XS Plus, we wanted to get more insight into the issues how interchangeable the INR results by CoaguChek XS Plus and STAR-R are and whether the INR results by CoaguChek XS Plus are reliable enough for monitoring the patients without being confirmed with the laboratory-based test. We found that INR values measured by CoaguChek XS Plus exhibited positive bias as INR values increased. Our results are in line with the previous findings that showed an increased INR difference at higher INR values [10, 18]. In addition to the overall correlation and agreement, we further compared the INR results in the lower INR (1.0-2.0 INR) and higher INR (2.0-3.0 INR) ranges and found profound difference or bias even in the therapeutic INR range (Figures 2 and 3).

Whether POCT INR measurement should be confirmed by the laboratory method or not is still debatable. Some studies insisted that the CoaguChek XS Plus is a reliable tool and dosing decision for vitamin K antagonist therapy may be safely made based on its INR results [13, 16]. On the contrary, Celenza and Skinner [15] concluded that although POC INR testing is sufficiently accurate to exclude clinically significant coagulopathy, laboratory-based INR measurements are still required to confirm nonnormal POC INR results, particularly in the supratherapeutic range. One study performed in the setting of anticoagulation clinic also showed that 33% (17/52) of INR measurements with the CoaguChek XS Plus was sufficiently different from the Stago-measured INR values to have resulted in a different therapeutic decision [10]. Noticeably, in the present study, 21/118 (17.8%) INR measurements showed a difference in dosing decision for the anticoagulation therapy between CoaguChek XS Plus and STA-R. Statistically, the agreement between the two instruments was not excellent but just fair to good, with kappa value of 0.679 (95% CI, 0.569–0.790) (Table 1). Our data supports the finding by Donaldson et al. (2010) and implies that dosing decision based on the INR results by CoaguChek XS Plus would be different from that based on the laboratory-based INR results.

In summary, we compared the PT INR results generated by the CoaguChek XS Plus and the STA-R to know how comparable the results would be in the professional hospital setting. This study is limited in that the sample size in the >3.0 INR range was very small. Nevertheless, we observed that the positive bias of CoaguChek XS Plus was obvious even in the therapeutic INR range and dosing decision based on the INR results by CoaguChek XS Plus would be different from that based on the INR results by STA-R. Our data does not support the assumption that POCT coagulometers can be used reliably and safely without being validated with the standard laboratory INR measurements. Even though the use of POCT coagulometers is getting increased in the professional setting, the INR measurements by POCT coagulometers, especially higher INR measurements, still need to be confirmed regularly with the laboratory INR measurements. Further studies with larger sample size and broad INR values would be necessary to confirm our findings.

## Figures and Tables

**Table 1:** Agreement of INR measurements between CoaguChek XS Plus and STA-R.

	CoaguChek XS Plus			Total (%)
	INR < 2	INR 2.0-3.0	INR > 3
STA-R				
INR < 2	65	13	0	78 (66.1)
INR 2.0-3.0	1	31	6	38 (32.2)
INR > 3.0	0	1	1	2 (1.7)

Total (%)	66 (55.9)	45 (38.1)	7 (5.9)	118

Kappa value was 0.679 (95% CI, 0.569–0.790).

**Table 2:** Previous studies on the performances of CoaguChek XS Plus system.

Study	Number of measurements	Setting	Laboratory-based system	Comparison using Bland-Altman plot, mean bias (95% limit of agreement)
Deom et al. (2009) [13]	259	Community setting (38 doctor's offices)	Innovin (Siemens/Dade-Behring)	0.03 INR (−0.67 INR ~ 0.74 INR)
Urwyler et al. (2009) [14]	227	Perioperative setting	Innovin (Siemens/Dade-Behring)	2.3% (95% CI = −20.3% ~ 24.9%)
Donaldson et al. (2010) [10]	52	Pharmacist-managed anticoagulation clinic	Stago (Diagnostica-stago)	0.27 INR (−0.33 INR ~ 0.88 INR)
Celenza and Skinner (2011) [15]	293	Emergency department	Not specified	−0.0 INR (−1.1 INR ~ 1.1 INR)
Lawrie et al. (2012) [16]	168 for innovin and 115 for PT-Fib HS+	Overanticoagulated (INR > 4.5) patients in anticoagulation clinic	Innovin (Siemens/Dade-Behring) on CA-7000 analyzer (Sysmex) and PT-Fib HS+ (Instrumentation Laboratory) on CA-1500 analyzer (Sysmex)	Innovin: −0.1% (−22.0% ~ 22.1%) PT-Fib HS+: 8.2% (−21.4% ~ 37.9%)
Urwyler et al. (2012) [17]	73	Pediatric intensive care unit setting	Innovin (Siemens/Dade-Behring)	1.22% (−27% ~ 56%)

Abbreviations: INR: international normalized ratio; SD: standard deviation; CI: confidence interval; PT-Fib HS+: HemosIL PT-Fibrinogen HS Plus.
